# Single Step Process for Crystalline Ni-B Compounds

**DOI:** 10.3390/ma11071259

**Published:** 2018-07-22

**Authors:** Mahboobeh Shahbazi, Henrietta Cathey, Natalia Danilova, Ian D.R. Mackinnon

**Affiliations:** Institute for Future Environments and Science and Engineering Faculty, Queensland University of Technology (QUT), Brisbane QLD 4001, Australia; mahboobeh.shahbazi@qut.edu.au (M.S.); henrietta.cathey@qut.edu.au (H.C.); natalia.danilova@qut.edu.au (N.D.)

**Keywords:** nickel boride synthesis, sodium borohydride, microprobe analysis, microstructure, autogenous pressure

## Abstract

Crystalline Ni_2_B, Ni_3_B, and Ni_4_B_3_ are synthesized by a single-step method using autogenous pressure from the reaction of NaBH_4_ and Ni precursors. The effect of reaction temperature, pressure, time, and starting materials on the composition of synthesized products, particle morphologies, and magnetic properties is demonstrated. High yields of Ni_2_B (>98%) are achieved at 2.3–3.4 MPa and ~670 °C over five hours. Crystalline Ni_3_B or Ni_4_B_3_ form in conjunction with Ni_2_B at higher temperature or higher autogenous pressure in proportions influenced by the ratios of initial reactants. For the same starting ratios of reactants, a longer reaction time or higher pressure shifts equilibria to lower yields of Ni_2_B. Using this approach, yields of ~88% Ni_4_B_3_ (single phase orthorhombic) and ~72% Ni_3_B are obtained for conditions 1.9 MPa < P_max_ < 4.9 MPa and 670 °C < T_max_ < 725 °C. Gas-solid reaction is the dominant transformation mechanism that results in formation of Ni_2_B at lower temperatures than conventional solid-state methods.

## 1. Introduction

**Figure 1:**
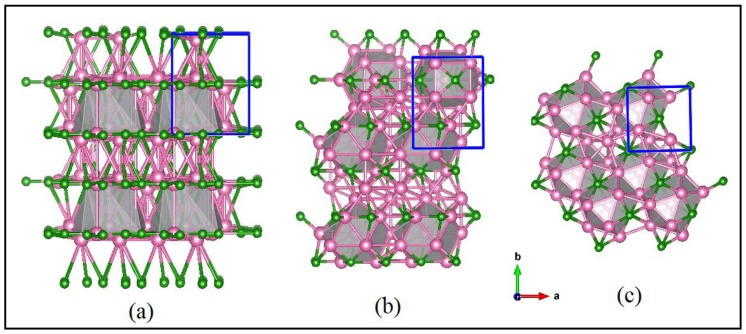
Crystal structures of (**a**) Orthorhombic Ni_4_B_3_, (**b**) Ni_3_B and (**c**) Ni_2_B. Unit cells are outlined in blue. Ni atoms are pink and B atoms are green.

Nickel boride compounds are useful industrial materials due to their microhardness, high chemical and thermal stability, excellent selectivity and activity for liquid phase reactions and their effectiveness as catalysts. The Ni-B system comprises several stable crystalline compounds for which their structures are well described in the literature: NiB, Ni_3_B, Ni_2_B, Ni_4_B_3_, Ni_7_B_3_, and Ni_23_B_6_ [1,2,3,4,5,6]. Examples of the three most common forms, orthorhombic Ni_4_B_3_, Ni_2_B, and Ni_3_B, are shown in Figure 1. Boron-rich nickel borides are paramagnetic while nickel-rich boride compounds are soft ferromagnets [3,7,8,9,10].

The functionality of Ni-B compounds is dependent on the form—amorphous or crystalline—often determined by the method of synthesis. For example, amorphous Ni-B compounds are highly active catalysts for hydrogenation and other organic reactions and are prepared by chemical reduction [11,12] using hydrolysis of NaBH_4_ with a metal salt [13,14]. Crystalline forms of Ni-B are determined by sintering temperatures and heating duration applied to a precursor material which may include amorphous Ni-B compositions [15]. A good example of functional dependence is recently described by Jiang et al. [4] who developed an efficient oxygen evolution reaction (OER) electrocatalyst using Ni_3_B nanoparticle cores surrounded by nickel borate shells [16]. In their work, the crystallinity of Ni_3_B shells is found to affect the OER electrocatalyst activity. Partially crystalline Ni_3_B shells exhibit a higher OER activity than the amorphous or fully crystalline counterparts [13].

Solid state synthesis of boron compounds is usually undertaken at high temperature due to the high melting point of boron. For example, Ni_3_B is synthesized at 1000 °C [4], Ni_2_B at 1200 °C [17] and Ni_4_B_3_ at 750–900 °C [5]. Several other lower temperature methods have been suggested for synthesis of nickel boride compounds including ball milling of the elements [18], boronizing of pure nickel [19], and a solvo-thermal route [20]. Schlesinger et al. [21] reported a simple synthesis of nickel boride using nickel salts and sodium borohydride, which results in the formation of a black precipitate with elemental ratios equivalent to Ni_2_B. Their work focused on NaBH_4_ hydrolysis with Co and the effectiveness of the product as an accelerator for the production of organic acids. As a result, structural data on the form of the black precipitate with Ni_2_B composition are not available [21].

The room temperature synthesis of a Ni-B compound via hydrolysis of metal borohydrides is also reported by Caputo et al. [15]. In both cases, the amorphous Ni-B precipitate requires sintering at >250 °C in ultrahigh purity argon for a minimum of 2 h to achieve a crystalline form [12,22,23]. Nanoparticles of nickel diboride (NiB_2_) have also been produced by reducing nickel iodide with lithium borohydride in anhydrous oxygen-free tetrahydrofuran (THF) [24].

In this article, we report the fabrication of Ni_2_B, Ni_3_B, and orthorhombic Ni_4_B_3_ (o-Ni_4_B_3_) compounds at moderate temperature and autogenous pressure using a Parr reactor. These syntheses utilize autogenous pressure derived from the decomposition of sodium borohydride in the presence of Ni. The optimum pressure attained via this process for Ni_2_B synthesis is ~2.3–3.4 MPa at a maximum temperature of ~670 °C. We describe the structure and microstructure of these compounds using X-ray diffraction and electron beam methods. The influence of reaction temperature, pressure time and starting materials on the composition of synthesized products, particle morphologies, and magnetic properties is demonstrated.

## 2. Materials and Methods

### 2.1. Boride Synthesis

Molar ratios of Ni (<150 μm; 99.9% purity) and NaBH_4_ powder (99.99% purity) supplied by Sigma-Aldrich (St. Louis, MO, USA) are weighed, gently ground in an agate mortar, and placed into a 50 mL Parr reactor within a controlled atmosphere glove box containing Argon (99.99% purity). Mixing of starting materials noted above does not approximate the well-known effects of ball-milling [18]. For some experiments, a boron nitride sleeve is introduced into the reactor to minimize reaction with Ni-rich side-walls of the reactor. Details of operational practices using this type of reactor are provided in earlier work [25,26].

The reactor design results in a thermal gradient of ±200 °C between the bottom and top of the reactor during ramp up to the equilibration temperature. An advantage of this reactor design is that gaseous phases such as sodium and sodium hydride condense at the cooler top of the reactor. On cooling the reaction chamber to room temperature, the reactor is opened in the argon-filled glove box *via* slow pressure equilibration using a gas release valve.

Since all starting materials are placed at the base of the reactor and heating elements are located at the base, we have calibrated the actual temperature at different measured heights with the thermocouple readings, which are made at the center of the reactor. Calibrations show that the relative error in temperature estimates at the reactor base is ±20 °C at ~400 °C. In this study, we use the calibrated temperatures for the base of the reactor. A consistent heating rate of 10 °C/min is used in all reactions, albeit at different temperatures, and the heating rate is held constant for varying periods of time. In general, the reactor heating rate is held constant to achieve temperatures at the reactor base of: (i) 140 °C; (ii) 420 °C; and (iii) target temperatures (i.e., T_max_) of 670 °C or 725 °C for variable periods of time. These specific heating rates and constant temperature periods are identified in Table 1.

### 2.2. Characterization

Polycrystalline samples are characterized using X-ray powder diffraction and electron microscopy equipped with microanalysis. X-ray powder diffraction patterns are obtained using Co and Cu Kα1 radiation in Bragg Brentano geometry with 0.02° 2θ steps and a counting time of 10 s per step using PANalytical X-ray diffractometers (Almelo, The Netherlands). Diffraction patterns are refined and indexed using the software program Topas (Florence, KY, USA) [27]. X-ray diffraction patterns and electron microscopy indicate that many synthesized samples are multiphase with Ni_2_B_,_ Ni_3_B, or Ni_4_B_3_ as a predominant phase. Samples with high proportions of Ni_2_B or Ni_3_B (i.e., >95%) are selected for additional data collection using a PANalytical X-ray diffractometer for subsequent Rietveld refinement using Topas. Schematic models of crystal structures shown in Figure 1 are compiled by the program VESTA [28].

A Zeiss Sigma^TM^ Field Emission Gemini SEM (Carl Zeiss Pty Ltd., North Ryde, Australia) equipped with an Oxford Instruments SDD XMax 50 mm^2^ detector (Abington, UK) is used for microscopy observations and energy dispersive spectroscopy (EDS) analysis. Samples are prepared for SEM/EDS by placing a thin layer of powder onto aluminum stubs with double sided carbon tape. In general, samples are not coated with a conductive coating to avoid analytical interference(s). Elemental analysis is carried out at an accelerating voltage of 15 kV at 8.5 mm working distance. Excessively charging samples are imaged at lower accelerating voltages of 5 kV or 10 kV.

Microstructural features were examined on polished samples using a field emission JEOL 7001SEM (JEOL (Australasia) Pty Ltd., French’s Forest, Australia), with automated feature detection equipped with secondary electron (SE, JEOL (Australasia) Pty Ltd., French’s Forest, Australia), Oxford Instruments SDD XMax 50 mm^2^ detector (Abington, UK) and an electron back-scatter diffraction (EBSD) pattern analyzer and Channel 5 analysis software. Fine polished samples mounted in conductive resin were selected for EBSD mapping using an accelerating voltage of 15 kV and step size of 0.5 µm.

Quantitative elemental analyses are performed using a JEOL JXA 8530F field emission electron microprobe analyzer (EPMA, JEOL (Australasia) Pty Ltd., French’s Forest, Australia) equipped with five wavelength-dispersive spectrometers and using *Probe for EPMA* software [25]. For these analyses, powder samples are mixed with conductive resin and placed in a 30 mm diameter mold inside a hot mounting press. The sample mount is polished with a series of diamond pads and cloths to a mirror finish suited to electron microprobe analysis. Spot analyses on borides are performed using the following combined conditions: B Kα X-ray intensities are measured at 7 kV accelerating voltage, followed immediately by intensity measurement of the Ni Kα X-ray line at 15 kV accelerating voltage. A focused beam is utilized and the beam current maintained at 40 nA under both conditions. Details on data collection methods, analyzing crystals, standard materials, and data reduction for EPMA analyses are provided in Shahbazi et al. [25].

In this study, the combined analytical conditions described above were chosen to localize the lateral and vertical spatial resolution from characteristic X-rays [25]. The detection limits under these conditions are 0.16 wt % for B and 0.04 wt % for Ni. The average analytical error for boron for individual spot analyses is 8.7%. A statistical program is used to determine the envelope for production of characteristic X-rays using the EPMA under these operating conditions. The lateral and vertical spatial resolutions for excitation of the Kα X-ray lines of B and Ni is <1 µm as calculated using the CASINO Monte Carlo modeling program for electron trajectories [25]. DC magnetization measurements are performed using a Cryogenics Ltd. Mini cryogen-free 5 T system (London, UK). The magnetization field isothermal loops are determined within ±5 T at 100 K.

## 3. Results

Data presented in this study are a summary of more than thirty separate experiments across a range of temperature and pressure conditions, and with different amounts and ratios of starting materials. We present a selection of these experimental data to demonstrate the range of conditions under which Ni-B compounds may form.

### 3.1. Synthesis

Selected reactions that illustrate the temperature and pressure conditions for production of Ni_2_B, Ni_3_B and o-Ni_4_B_3_ are listed in Table 1. In general, these reactions provide higher yield of nickel borides and, in some cases, single phase formation of Ni_2_B. In all reactions, the maximum pressure (P_max_) increases to between 1.9 MPa and 4.9 MPa when the reactor is at the maximum temperature. As shown in Table 1, the value of P_max_ varies for each reaction and is dependent on a number of variables including the ratio and amount of starting materials within the reactor. In general, a higher proportion and amount of NaBH_4_ in the reaction results in a higher P_max_.

**Figure 2:**
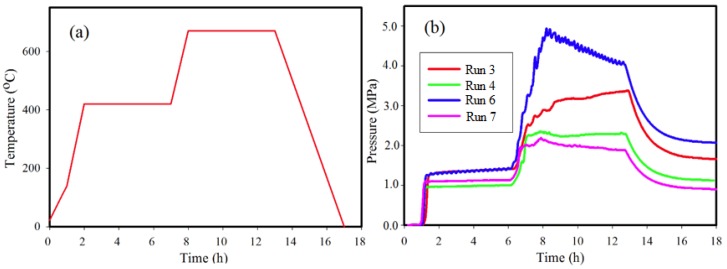
(**a**) Heating profile and (**b**) pressure profile for synthesis of Ni-B phases for Run 3, Run 4, Run 6, and Run 7 with conditions as listed in Table 1.

Figure 2a illustrates the typical heating profile used for Runs 3, 4, 6, and 7 and is the general format for all other reactions not listed in Table 1. The pressure profiles for some reactions listed in Table 1 are shown in Figure 2b. The maximum pressure achieved during a reaction depends on the quantity of reactants, in particular of NaBH_4_, used in the reactor [25]. For all reactions, pressure increases to a maximum value (P_max_) ranging between 2.3 MPa and 4.9 MPa at 670 °C. A rapid increase in pressure occurs as the thermocouple records a ramp from ~140 °C to 425 °C and also from 425 °C to 670 °C. The fluctuations in pressure profiles shown in Figure 2 are points in the reaction at which phase transformations are rapidly occurring as noted in earlier work [26,29]. Similar pressure profiles are observed for all other reactions listed in Table 1.

X-ray diffraction (XRD) and Rietveld refinements show that Ni_2_B is the major phase for Run 3, Run 4, and Run 5 with maximum yields of 99%, 98%, and 92%, respectively (Table 1). XRD peaks corresponding to boron are not observed for Run 3 in which there is an excess of boron in the reactants compared with the stoichiometric product. This outcome may be due to a higher P_max_ compared with Run 4 and Run 5 or formation of gaseous phases that condense at the top of the reactor. However, an increased reaction time to 40 h at 670 °C (Run 5) shows a reduction in Ni_2_B and a detectable quantity of Ni_3_B.

A reaction at significantly higher P_max_, as in Run 6 (Figure 2b and Table 1) achieved by increasing the amount of starting materials, results in substantially different proportions of Ni_2_B and Ni_3_B at 61% and 39%, respectively. The autogenous pressure for Run 6 is at a maximum of 4.9 MPa at T_max_ and may influence the formation of Ni_3_B for starting compositions that are deficient in Ni. For stoichiometric amounts of starting materials (Runs 7 and 8; Table 1), a relatively minor increase in P_max_ also shows a higher Ni_3_B yield albeit with residual Ni present. In comparison, Runs 8 and 9 show that with continued heating at 670 °C for a longer time, up to 15 h, the proportions of Ni_3_B to Ni_2_B shift in favor of increased Ni_2_B production and decreased Ni_3_B.

### 3.2. Structural Analysis

**Figure 3:**
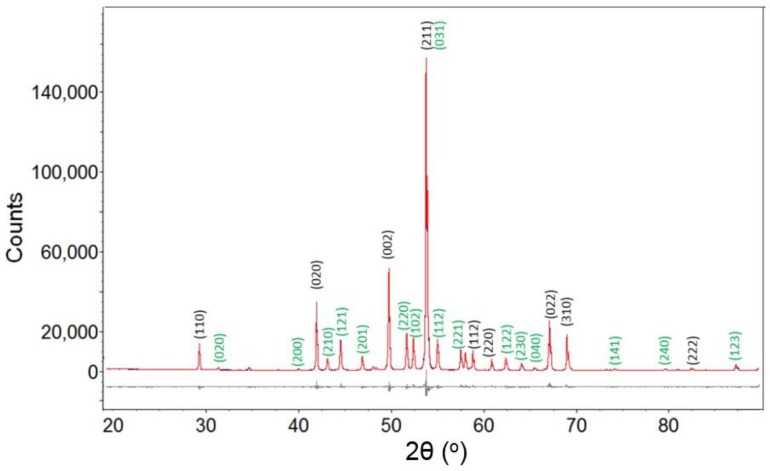
The experimental (blue), fitted (red) and difference (grey line below observed and calculated pattern) X-ray diffraction (XRD) profile for sample from Run 5. Indexed reflections for Ni_2_B and Ni_3_B are indexed with black and green color, respectively.

Powder diffraction patterns from selected samples listed in Table 1 are used for Rietveld refinement using Topas. Structural data from Kayser et al. and Havinga et al. [30,31] are used as input for refinement of XRD data for these samples. In the Ni-B system, peaks for Ni, o-Ni_4_B_3_, Ni_2_B and Ni_3_B are identified in XRD powder diffraction patterns. For reactions with more than one phase in the product, for example Run 5 (Table 1), refinements include allocation of diffraction peaks to each phase and unconstrained Rietveld refinement on all phases. As shown in Figure 3, Ni_2_B and Ni_3_B are readily distinguished using Rietveld refinement and visual inspection of peak intensities verifies that Ni_2_B is the predominant phase. Table 2 shows refined lattice parameters for Ni_2_B and Ni_3_B for Run 4 and Run 8 from this study, respectively. The refined cell parameters are in good agreement with results from Kayser et al. and Havinga et al. [15,16] with good quality refinement parameters such as goodness-of-fit.

### 3.3. Morphology and Microstructure

**Figure 4:**
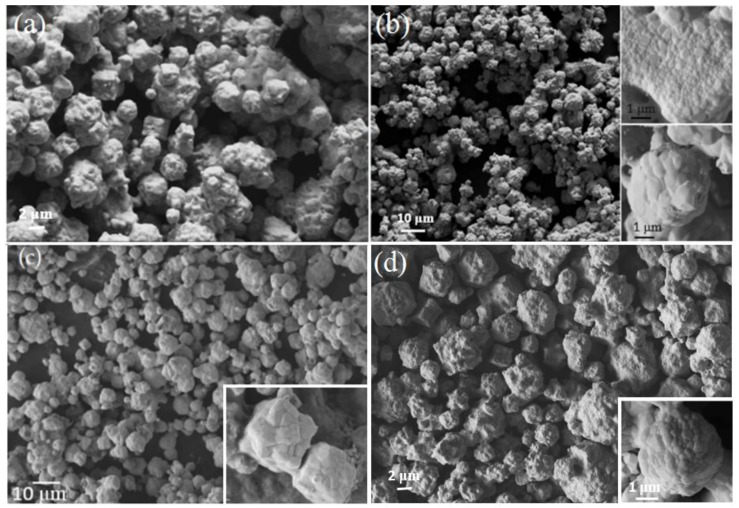
Scanning electron microscope (SEM) image of Ni-B powder from (**a**) Run 3, (**b**) Run 4, (**c**) Run 6, and (**d**) Run 8. Higher resolution SEM image shows (**c**) a cube and (**d**) sphere shaped morphology consisting of smoothly rounded crystals.

The morphology of Ni-B powders obtained from Run 3, Run 4, Run 6, and Run 8 are shown in Figure 4. The product is comprised of large aggregates with a broad distribution of many smaller particles. The Ni-B powders obtained at 670 °C are large aggregates consisting of well-defined, inter-penetrating polygonal particles with orthogonal morphology. In all samples produced by these syntheses, two different morphologies of aggregates are common; each of a size ranging from 2 μm up to 10 μm. These two morphologies are orthogonal or rectangular shaped and spherical shapes as shown in the insets of Figure 4c,d. Each aggregate morphology shows smooth surfaces with individual particles of size varying from 50 nm up to several hundred nm in size.

**Figure 5:**
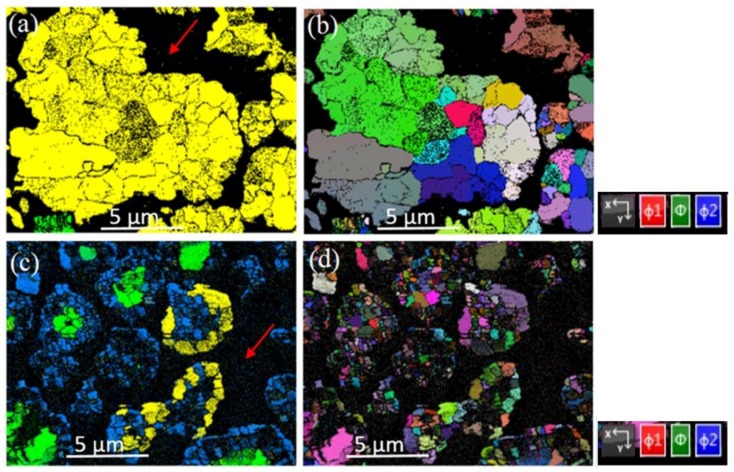
Electron back-scatter diffraction (EBSD) images of polished surfaces for Ni-B aggregates from Run 3 and Run 8. Red arrows indicate mounting resin. (**a**) EBSD map of the sample from Run 3 showing predominantly Ni_2_B (yellow) with minor Ni_3_B (green); (**b**) Euler map of the aggregate from Run 3 with polar orientations (right hand panel); (**c**) EBSD map of sample from Run 8 with three phases including Ni_2_B (yellow), Ni_3_B (blue) and Ni (green). (**d**) Euler map of the aggregates from Run 8.

EBSD analyses of Ni-B aggregates from Run 3 and Run 8 are presented in Figure 5 without the use of noise reduction software applied to the maps. Figure 5a indicates Ni_2_B is a major phase in the sample and confirms XRD data that ~99% of the Run 3 sample is Ni_2_B. A very small region in Figure 5a shows also the presence of Ni_3_B in green. Different microstructures were obtained for aggregates from Run 8, as illustrated in Figure 5c. According to the analysis using EBSD, three phases including Ni_2_B, Ni_3_B, and Ni are present in this sample. This determination is in good agreement with the XRD results for this sample (Table 1). Figure 5b,d clearly indicate that Ni_2_B grains produced in Run 8 are significantly smaller than those produced in Run 3 due to different reaction conditions. An Euler map in Figure 5b suggests that small Ni_2_B grains within the aggregate show a limited number of different orientations compared with the sample from Run 8 shown in Figure 5d. Texture maps can be used to evaluate grain orientation in these polished samples. For Run 3, these maps (not shown) show that preferential grain orientation in these aggregates is along the [001] and [110] primary axes for Ni_2_B grains.

**Figure 6:**
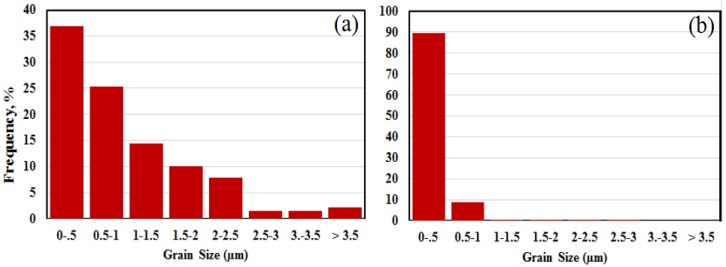
(**a**,**b**) Grain size distributions from Run 3 and Run 8, respectively, determined using EBSD data as shown in Figure 5.

Figure 6 uses the EBSD phase maps in Figure 5 to estimate the grain size distribution within an aggregate. For each analysis of Run 3 and Run 8 samples, approximately 300 grains are included in the dataset. The grain size distribution for Run 3 is substantially broader than that of aggregates in Run 8. Both graphs in Figure 6 show that the highest proportion of grains are sub-micron in size and that for Run 8 almost all grains are <1 µm.

**Figure 7:**
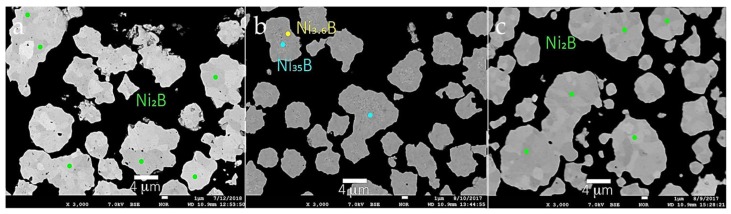
Backscattered electron images of aggregates from (**a**) Run 3; (**b**) Run 4; (**c**) Run 5. Colored circles denote locations of spot analyses using the EPMA coded to compositions listed in the figure.

Figure 7 shows backscattered electron images (BEIs) of aggregates from Run 3, Run 4, and Run 5. These images are used as a guide to identify locations for EPMA analyses and, in general, reflect differences in atomic number contrast within each aggregate. Table 3 provides average compositions from selected spot analyses of different aggregates from Runs 3–5. The number of point analyses used for average value determinations based on similar BEI contrast are listed in Table 3. Standard deviations are calculated on a basis of the number of analyses for each region within aggregates. These data show that the concentration of boron ranges between 0.52 wt % to 8.26 wt % and the concentration of nickel between 91.8 wt % and 99.3 wt % in the aggregates measured. As shown in Table 3, the stoichiometry of Ni-B phases include Ni_2_B and Ni_3_B in Run 3, Run 4, and Run 5. The stoichiometry in each case is based on an integer value for boron.

However, for Run 4, a Ni-rich core and a Ni-B outer shell is observed and shown in Figure 7b. The inner region of this aggregate shows an average composition of Ni_35.1_B, which was not observed by XRD. This composition may represent an unusual form of nickel boride, or alternatively, very fine-grained Ni metal particle(s) may be present in the core region of this grain. Detection of this Ni-rich region is due to the spatial and analytical resolution of the XRD vs. EPMA techniques. For example, XRD is a bulk technique that cannot detect phases present in amounts less than ~5%. However, the detailed internal structures of individual particles that have a two-phase or multi-phase core-shell structure that evades detection by XRD are readily identified using BSE imaging at high magnification. Such core and shell compositions, respectively, can be analyzed by WDS on a field emission electron microprobe (FE-EPMA), which permits high precision micron to sub-micron scale quantitative analysis. The interface region of an aggregate in this sample shows an average composition of Ni_3.4_B. These compositions are different to the stoichiometry of the starting materials.

EPMA analysis for aggregates from Run 3 with the Ni:B ratio of 4:3 shows an average composition of Ni_2.1_B and a fewer number of grains with Ni_3.20_B composition. This latter composition is in general agreement with observation of a minor Ni_3_B phase using EBSD analysis (Figure 5a). However, peaks from Ni_3_B were not observed in the XRD diffraction pattern from Run 3. The starting materials in Run 3 have excess boron compared with other reactions listed in Table 1. The stoichiometry for samples with Ni_2.22_B may be re-expressed as Ni_6.67_B_3_, which is similar to the phase Ni_7_B_3_ of hexagonal symmetry in space group P6_3_mc [3] However, XRD data does not support an hexagonal structure for this predominant product from Run 3. More importantly, XRD data and results from EBSD analysis show that Ni_2_B is the predominant phase from Run 3.

### 3.4. Magnetic Properties

**Figure 8:**
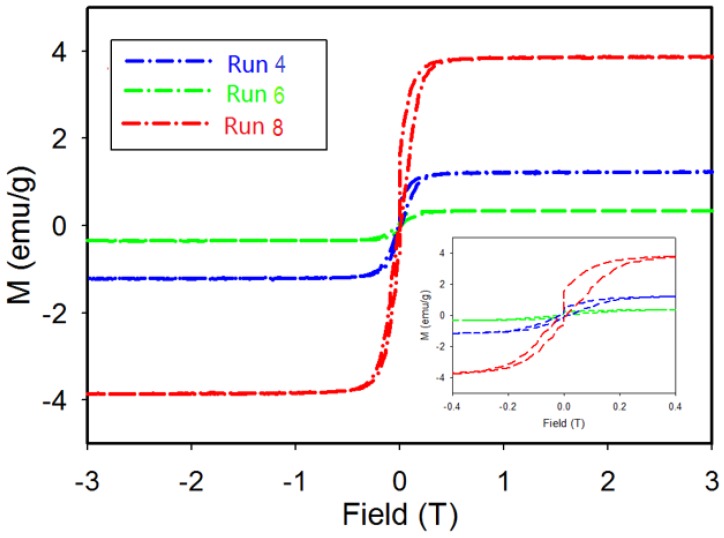
Field dependent magnetization at 100 K for samples from Run 4, Run 6, and Run 8. Inset: Detailed view of magnetization at low field.

The magnetization as a function of the applied field is determined for products from Run 4 (Ni_2_B-98%), Run 6 (Ni_2_B-61%, Ni_3_B-39%), and Run 8 (Ni_2_B-17%, Ni_3_B-72% and Ni-10%) at 100 K. As shown in Figure 8, the M-H curve for product from Run 4 with nearly single phase Ni_2_B displays a hysteresis curve with low magnetization values along with an increase in magnetization at low magnetic fields. The sample then saturates at an applied field of 0.15 T (inset). The magnetization data for products from Run 8 reveal a hysteresis loop with a significant increase in M value along with saturation at 0.25 T. This behavior could be due to the presence of two magnetic components; i.e., a ferromagnetic component such as Ni that is easily saturated at low field and a paramagnetic component such as Ni_3_B and/or Ni_2_B compounds. This interpretation is in agreement with results from XRD, EBSD, and EPMA analyses presented in earlier sections.

EPMA results for the sample from Run 4 show that areas with a nickel rich core are present and are identified in Figure 7b. The inclusion of nickel-rich phases (such as Ni_35.1_B or Ni) may result in a minor hysteresis effect in magnetic measurements. Magnetic measurement of the product from Run 8 with a dominant Ni_3_B phase and 10% Ni—as indicated by XRD results—reveals a larger hysteresis loop due to the higher amount of Ni present. The lower value of magnetization for the product from Run 4 is consistent with the known weak paramagnetic behavior of Ni_2_B [33]. The magnetization measurement for Run 6 displays low magnetization values along with an increase in magnetization at a low magnetic field and then saturation at an applied field of 0.2 T. Unlike the other two samples, hysteresis is not observed for this sample from Run 6. In this case, the absence of Ni or Ni-rich particles is consistent with the observed hysteresis behavior of the sample.

## 4. Discussion

In general, syntheses of Ni-B compounds are based on (i) solid-state methods that include mechanical mixing of components followed by heating and sintering protocols under reducing conditions or (ii) solution based methods that involve precipitation of precursor components that are, preferably, intimately mixed at nano-scale followed by heating and sintering under reducing conditions. In the latter case, if the mixture of components is effective and intimate, then the achievement of a specific stoichiometric composition of Ni-B compound can be achieved at a lower temperature than solid-state methods.

The production of metal borides via room temperature hydrolysis of NaBH_4_, sometimes catalyzed by a salt, is well known [21]. More recently, metal borides have been produced with organic compounds such as tetraethyleneglycol (TEG) [23] and tetrahydrofuran (THF) as solvent [15]. In these instances, it is critical to thoroughly wash the resultant precipitate, which may be crystalline [15] or amorphous [14] in order to remove remnant salts or solvents. These requirements and the potential for production of mixed phases [23] suggests that single step syntheses of a stoichiometric nickel boride by solution based methods are unlikely. Nevertheless, this polyol or modified polyol approach can result in the direct formation of nanocrystalline metal borides without a requirement for post-synthesis annealing [23].

In this work, we explore the potential for single step synthesis of crystalline nickel borides using the known reactivity of NaBH_4_ on heating with specific metals. The capacity for NaBH_4_ to generate gases (e.g., B_2_H_6_ and/or H_2_) via pyrolysis in the presence of specific metals was identified in the 1950s [34]. We have exploited this characteristic of NaBH_4_ and KBH_4_ in order to form MgB_2_ [26,29] and Mg-Ni borides [25] under similar reactor conditions. A consistent heating protocol is utilized for all experiments albeit with variations in the hold time at maximum temperature of reaction. Autogenous pressure is moderated by the ratio of reactants (e.g., Ni to NaBH_4_) and the volume of NaBH_4_ in the starting mixture. As shown in Table 1, the ratio of reactants also influences the optimal stoichiometry of a final product.

### 4.1. Synthesis of Nickel Borides

Different nickel boride phases can be produced at moderate temperatures by heating Ni and NaBH_4_ under autogenous pressure generated by the decomposition of the borohydride. The general chemical equation that describes this reaction is:

(1) $$2{\mathrm{NaBH}}_{4}(s)\;+\;x\mathrm{Ni}(s)\;\to \;x\sum_{i}{\left(Ni,B\right)}_{i}\left(s\right)\;+\;2\mathrm{NaH}(s)\;+\;3H2(g)$$

where (*Ni, B*)*_i_* stands for any possible binary solid phase formed by Ni and B. In this generic case, different reaction paths may occur and the exact mass balance of a specific reaction will depend on the ratio of starting materials as well as the number of nickel boride phases formed and their resultant stoichiometry. According to (1) above, the maximum amount of gas produced from the decomposition of NaBH_4_ is three moles of H_2_ for every two moles of NaBH_4_. In general, this trend is observed across the full range of experiments as well as those presented in Table 1. However, at the temperatures achieved in these experiments other hydrides, such as NaH, may also form as a gaseous species [26].

The optimum conditions for formation of Ni_2_B using NaBH_4_ and Ni metal are T_max_ = 670 °C and 2.3 MPa < P_max_ < 3.4 MPa with maximum yield of 98–99%, determined by XRD as shown in Table 1. Orthorhombic Ni_4_B_3_ forms at higher temperature (~725 °C) than Ni_2_B under these conditions (i.e., 1.9 MPa < P_max_ < 2.8 MPa) with yield up to 88% and at a lower pressure. Our assumption that a longer hold time at 725 °C would reap a higher yield of o-Ni_4_B_3_ did not materialize despite a number of attempts (data not shown) and suggests that the phase diagram under these conditions is complex, particularly given the substantial presence of Ni_2_B in Run 2 at pressures overlapping with those achieved in Runs 3 and 4.

Figure 2 shows the autogenous pressure changes with reaction for both Run 3 and Run 4. For both reactions, the autogenous pressure remains relatively constant during the hold at T_max_ (i.e., ~670 °C) unlike the pressure profile for Run 6. The higher pressure achieved in Run 3 compared to Run 4 is due to a higher amount and proportion of NaBH_4_ in the reactant mix, yet allows an estimate of the boundary conditions for formation of Ni_2_B. The profiles for Run 3 and Run 4 at T_max_ suggest that the optimum equilibrium conditions for formation of Ni_2_B using autogenous pressure are achieved with a resultant high yield. The outcome from Run 5, which is a repeat of Run 4 but for a longer hold time (8x longer) at T_max_, suggests that Ni_3_B may be a preferred stable phase at this temperature and pressure.

In all cases shown in Figure 2, autogenous pressure increases rapidly as the reaction temperature increases to 420 °C. We have shown in previous work [25,26,29] that this rise in autogenous pressure is due to formation of H_2_ and B_2_H_6_ by the decomposition of NaBH_4_. This decomposition of NaBH_4_ increases rapidly with temperatures above 400 °C and will depend on the relative proportions of reactants, pressure, temperature and heating rates. In Run 6 (Figure 2), the pressure is the highest of those listed due to the higher total amount of reactants and with proportionately more NaBH_4_ than in other runs. The irregular changes in pressure and the significant drop in autogenous pressure (Δ~1 MPa) during the hold at T_max_ for Run 6 suggests that under these conditions, Ni_2_B and Ni_3_B are competing for the available resource—boron—to form a stable phase. As B_2_H_6_ is used in the reaction, H_2_ remains and provides the ambient pressure once T ~ 25 °C at the end of the reaction. The competing reactions at T_max_ are (3) and (4) as shown below:

2NaBH_4_ = 2NaH + B_2_H_6_ (2)

2Ni + 2B_2_H_6_ + NaH = Ni_2_B + 3BH_3_ + 2H_2_ + Na (3)

3Ni + 2B_2_H_6_ + NaH = Ni_3_B + 3BH_3_ + 2H_2_ + Na (4)

Reactions (3) and (4) are balanced equations for a closed system but the detail of processes occurring in these reactions is difficult to verify. As noted in earlier work [26], the decomposition of NaBH_4_ may occur at low temperatures (<200 °C) in the presence of a metal catalyst [29] and accelerates at temperatures above ~400 °C *via* formation of lattice defects resulting in the production of BH_3_ and NaH [35]. We surmise that formation of BH_3_ gas which readily polymerizes to form B_2_H_6_ and higher forms [36] is promoted under the conditions inherent in the reactor. In addition, the melting point for NaH is 300 °C and the melting point for NaBH_4_ is 505 °C at standard pressure [37]. However, the melting point for NaBH_4_ is shown to decrease in the presence of metals [38,39] but may increase to 535 °C under 0.1 MPa hydrogen pressure [38]. Other factors that influence the intermediate reactions between ~400 °C and 675 °C include the formation of liquid Na and the rapid increase in vapour pressure of Na to 0.089 kPa above 420 °C at 0.1 MPa [40]. Hence, we offer reactions (3) and (4) above as possible pathways for the formation of Ni_2_B and Ni_3_B under these reactor conditions.

The data for Runs 7 and 8, when compared with Run 6, show that a higher proportion of Ni in the reaction mix results in a shift in the equilibrium between Ni_2_B and Ni_3_B at lower autogenous pressure (i.e., ~2.2 MPa). In addition, a longer hold time at ~670 °C results in a higher relative yield of Ni_3_B. In both cases, excess Ni is present in the reactants which we presume to be at the expense of Ni_2_B formation. As shown in Run 9, a longer hold time at ~670 °C for up to 15 h shifts the reaction back in favour of Ni_2_B, and outlines the limits of the phase field for formation of Ni_2_B and Ni_3_B under these conditions.

X-ray diffraction experiments by Kapfenberger et al. [14] under ambient atmosphere conditions indicate that crystalline Ni_3_B forms at about 320 °C. Other syntheses, such as the modified polyol process using TEG or mechanical alloying also indicate that Ni_3_B forms at lower temperatures than Ni_2_B. For example, the lowest temperature for Ni_3_B formation using a TEG polyol process is ~265 °C but above 400 °C Ni also forms in the product [23]. Similarly, with mechanical alloying Ni_3_B forms at a lower temperature (~350 °C) than Ni_2_B (~700 °C) [41]. While these synthesis methods are not directly comparable, we surmise that formation of Ni_3_B at lower temperatures than Ni_2_B from a given starting composition is consistent with the data presented in this work using autogenous pressure pyrolysis.

Ni_2_B is stable up to high pressures at ambient temperature [42] but is less amenable to solvo-thermal methods of synthesis. Feng et al. [20] produced Ni_2_B at ~420 °C with 60% yield using benzene as the solvent. Other methods, such as with an RF thermal plasma produce Ni_2_B from Ni and B precursors but in a multi-phase mix with Ni_3_B and Ni_4_B_3_. The highest yield of Ni_2_B by this process is ~28% [43]. Conventional solid state methods to synthesize Ni_2_B using Ni and B powders heated to temperatures >850 °C in an enclosed capsule [17] is a well utilised approach to obtain single phase product. In comparison, the use of autogenous pressure generated by heating NaBH_4_ in the presence of Ni powder generates predominantly single phase Ni_2_B at lower temperature as shown by powder XRD in Table 1. The presence of minor quantities of other phases such as Ni_3_B (see Table 3) is only detectable *via* more spatially precise methods such as EPMA. We propose that phase pure (i.e., 100% yield) Ni_2_B using this method can be achieved by fine-tuning experimental parameters such as the ratio of reactants, the reaction temperature profile and T_max_.

### 4.2. Magnetic Properties

The magnetic properties of transition metal metalloid crystalline compounds are very similar to amorphous counterparts with the same composition [1,7,8,10,44]. Gelatt et al. [45] report that the magnetic properties of the Ni-B system are governed by both B p orbitals and Ni d orbital–B p orbital interaction [45]. The B–B zigzag chains expand through NiB structures with increased B content. As the Ni content is diluted by boron, the Ni d orbital bonding becomes weaker. However, d states interact with metalloid s and p states, forming bonding and antibonding hybrids which can be more effective in bonding than the parent states. These bonding mechanisms strongly affect the formation of ferromagnetic states [46].

The M-H curve for the product from Run 4 with very high yield of Ni_2_B displays a hysteresis curve with low magnetization values along with an increase in magnetization at low magnetic fields. As shown in Figure 8, the magnetization saturates at an applied field of 0.15 T (inset). On the other hand, the product(s) from Run 8, with high Ni_3_B content shows a hysteresis loop with a significant increase in M value and saturation at 0.25 T (Figure 8). This behaviour is due to the presence of a ferromagnetic component (e.g., Ni) that easily saturates at low field and the presence of paramagnetic components such as Ni_3_B and Ni_2_B. As shown in Figure 5c, the product from Run 8 contains Ni particles embedded in paramagnetic Ni_3_B and Ni_2_B matrixes.

Thus, in general, our results from XRD, EBSD and EPMA analyses are consistent with high sensitivity magnetic measurements of these samples. While it is uncommon to present data on mixed phase materials, the magnetic properties shown in Figure 8 demonstrate that low levels of ferromagnetic material(s) can also be reliably detected by this technique. The higher magnetization value and broader hysteresis loop for the sample from Run 8, compared with Run 4 and Run 6 shown in Figure 8, is due to the higher Ni content as confirmed by XRD Rietveld analysis. The lower values of magnetization for products from Run 4 and Run 6 are also consistent with the weak paramagnetic behaviour of Ni_2_B [33,46] and the relative proportions determined by XRD. The absence of Ni in Run 6 product is also consistent with an absence of hysteresis in the data shown in Figure 8.

## 5. Conclusions

Crystalline Ni_2_B, Ni_3_B and o-Ni_3_B_4_ compounds have been synthesised at moderate temperature and autogenous pressure using a single step process. These syntheses utilise autogenous pressure derived from the decomposition of sodium borohydride in the presence of Ni. The optimum pressure attained *via* this process for high yield Ni_2_B synthesis is ~2.3–3.4 MPa at a maximum temperature of ~670 °C. The molar ratio of sodium borohydride to nickel powder affects not only the granularity and crystallinity of boride products but also the final composition. Spatially and chemically precise EPMA analyses show that boron content ranges from 0.5 to 8.5 wt % and nickel content from 91.7 to 99.3 wt % in the samples measured. This autogenous pressure method produces Ni_2_B in high yield and at lower temperatures than solid state methods.

## Figures and Tables

**Table 1:** Synthesis conditions for Ni-B compounds.

Run No.	Weight	Reactant Ni:B Ratio	T_Max_ (°C)	t_Tm_ (hours)	P_Max_ (MPa)	Compounds in Product
1	Σ = 5.37 g	1:2	725	5	1.9	Ni_4_B_3_ (o; 88%) (Ni_3_B)
2	Σ = 5.37 g	1:2	725	12	2.8	Ni_4_B_3_ (o; 46%) Ni_2_B (54%)
3	Σ = 3.48 g	4:3	670	5	3.4	Ni_2_B (99%)
4	Σ = 3.10 g	2:1	670	5	2.3	Ni_2_B (98%)
5	Σ = 3.10 g	2:1	670	40	2.5	Ni_2_B (92%) Ni_3_B (8%)
6	Σ = 6.22 g	2:1	670	5	4.9	Ni_2_B (61%), Ni_3_B (39%)
7	Σ = 3.74 g	3:1	670	5	2.2	Ni_3_B (55%), Ni_2_B (39%) Ni (6%)
8	Σ = 3.74 g	3:1	670	10	2.4	Ni_3_B (72%), Ni_2_B (17%) Ni (10%)
9	Σ = 3.74 g	3:1	670	15	2.3	Ni_3_B (64%), Ni_2_B (26%) Ni (10%)

**Table 2:** Rietveld refinement data for Ni_2_B and Ni_3_B from Run 5 and Run 9.

Formula	Ni_3_B (This Work)	Ni_3_B [6]	Ni_2_B (This Work)	Ni_2_B [32]
Crystal System	Orthorhombic (Pnma)		Tetragonal (I4/mcm)	
a(Å)	5.2273 (1)	5.2195 (5)	4.9768 (1)	4.9910 (3)
b(Å)	6.6131 (1)	6.6164 (6)	4.9768 (1)	4.9910 (3)
c(Å)	4.3900 (1)	4.3912 (4)	4.2348 (0)	4.2470 (3)
Cell Vol. (Å^3^)	151.756 (3)	151.65	104.891 (2)	105.79
R_wp_	5.4		3.86	
R_p_	3.86		2.61	
GoF	2.57		1.81	

**Table 3:** Average compositions of selected regions in aggregates from Run 3, Run 4, and Run 5.

Compound	*n*	Element wt %			Stoichiometry		
**Run 3**		**B**	**Ni**	**Totals**	**B**	**Ni**	**Totals**
Ni_2_B	7	8.1 (0.3)	92.4 (0.7)	100.5	1	2.1 (0.1)	3.1 (0.1)
Ni_2.2_B	3	7.6	91.7 (0.3)	99.3 (0.3)	1	2.22 (0.02)	3.2 (0.02)
Ni_3_B	2	5.4	94.3	99.8	1	3.20	4.2
**Run 4**							
Ni_2_B	15	8.2 (0.2)	91.9 (0.3)	100.1 (0.4)	1	2.07(0.06)	3.1 (0.1)
Ni_3_B	7	5.5 (0.3)	94.3 (0.5)	99.7 (0.3)	1	3.19(0.21)	4.2 (0.2)
Ni_3.5_B	4	4.9 (0.1)	94.6 (0.2)	99.5 (0.2)	1	3.53 (0.09)	4.5 (0.1)
**Run 5**							
Ni_2_B	12	8.3 (0.3)	92.2 (0.3)	100.5(0.5)	1	2.04(0.07)	3.0 (0.1)
Ni_3_B	5	5.5 (0.1)	94.6 (0.4)	100.1 (0.4)	1	3.14(0.09)	4.1 (0.1)
